# An analysis of emerging food safety and fraud risks of novel insect proteins within complex supply chains

**DOI:** 10.1038/s41538-023-00241-y

**Published:** 2024-01-20

**Authors:** A. Traynor, D. Thorburn Burns, D. Wu, N. Karoonuthaisiri, A. Petchkongkaew, C. T. Elliott

**Affiliations:** 1 Institute for Global Food Security, School of Biological Sciences, Queen’s University of Belfast, Belfast, BT9 5DL Northern Ireland UK; 2 National Measurement Laboratory: Centre of Excellence in Agriculture and Food Integrity, Institute for Global Food Security, School of Biological Sciences, Queen’s University Belfast, 19 Chlorine Gardens, Belfast, Northern Ireland BT9 5DL UK; 3 National Center for Genetic Engineering and Biotechnology, National Science and Technology Development Agency, 111 Thailand Science Park, Phahonyothin Road, Pathumthani, 12120 Thailand; 4 International Joint Research Centre on Food Security (IJC-FOODSEC), 113 Thailand Science Park, Phahonyothin Road, Khong Luang, Pathum Thani 12120 Thailand; 5 School of Food Science and Technology, Faculty of Science and Technology, Thammasat University, 99 Mhu 18, Phahonyothin road, Khong Luang, Pathum Thani 12120 Thailand

**Keywords:** Zoology

## Abstract

Food consumption play a crucial role in human life, yet conventional food production and consumption patterns can be detrimental to the environment. Thus, research and development has been directed towards alternative proteins, with edible insects being promising sources. Edible insects have been recognised for their sustainable benefits providing protein, with less emission of greenhouse gas, land and water usage compared to sources, such as beef, chicken, and dairy products. Among the over 2000 known edible insect species, only four, namely yellow mealworm (*Tenebrio molitor)*, migratory locust/grasshopper *(Locusta migratoria)*, grain mould beetle, also known as lesser mealworm which is a larval form of *Alphitobius diaperinus* (from the family of Tenebrionidae of darkling beetles) and house cricket *(Acheta domesticus)*, are currently authorised in specific products through specific producers in the EU. The expansion of such foods into Western diets face challenges such as consumer barriers, gaps in microbiological and chemical safety hazard data during production and processing, and the potential for fraudulent supply chain activity. The main aim of this study was to map the supply chain, through interviews with personnel along the supply chain, coupled with searches for relevant publications and governmental documents. Thus, the main potential points of food safety and fraud along the edible insect supply chain were identified. Feed substrate was identified as the main area of concern regarding microbiological and chemical food safety and novel processing techniques were forecast to be of most concern for future fraudulent activity. Despite the on-going authorisation of insect species in many countries there are substantial food safety and authenticity information gaps in this industry that need to be addressed before edible insects can be viewed as a safe and sustainable protein sources by Western consumers.

## Introduction

### Is there need for another novel protein in our food and feed markets?

Food production and consumption plays a vital role in human life; however, consumers are becoming increasingly aware of the impacts of unsustainable production methods. Despite the challenge of feeding an estimated 10 billion people by 2050, conventional livestock industries have been criticised for their resource-intensive production methods^1^. Today’s consumers have the power to reconfigure the global food system through their demands for safe, sustainable and authentic food choices. Driven by consumer demands and market opportunities, research and development have been conducted on alternatives to meat protein products, with EY Food and Agriculture^2^ forecasting this industry to replace 5–10% of the global meat market by 2030, from less than 1% in 2020.

Owing to the nutritional and environmental advantages of entomophagy, research and opinions have pointed to edible insects as the most promising alternative protein sources. Entomophagy, the consumption of insects, currently takes place in over 120 countries world-wide, the majority in Asia and Africa^3,4^. However, recently, Western countries have developed interests in edible insects as potential sustainable protein sources. In line with consumer demand for sustainable and transparent products, the mass rearing of edible insects for feed and food has been forecast to exponentially increase over the next few years, as a sustainable alternative to resource-intensive meat protein production^4,5^.

Edible insects exhibit many sustainability and nutritional advantages compared to conventional animal protein sources^6^. Rumpold & Schülter^7^ defines insect proteins as, a ‘complete source of high-quality proteins’ in addition to a good source of fats, fibre, vitamins and minerals such as iron and calcium. They can be consumed at different life stages such as eggs, larvae and adults, adding to their potential as a sustainable protein source^8^. Moreover, vertical farming, whereby insects are reared in stacked cultivation layers, maximises production per square metre and reduces the demand for arable land. Some studies have reported up to ten times greater yield vertically farmed as opposed to traditional farming methods and less water usage^9–11^. Additionally, the ability of insects to utilise food waste streams and convert them into high quality protein reduces their ecological footprint by less water usage and emission of GHG’s^12^. At first glance, edible insects seem to be a simple solution to the challenge of food insecurity within a rising population, however, many of these sustainability claims are dependent on insect species, rearing conditions and processing.

The environmental sustainability of large-scale insect farming to include rearing, harvesting and production is largely unknown, and therefore makes it difficult to compare to the future sustainability of traditional livestock farming^13^. Despite increased efforts, there is an imperative need for more investigation and research into this area, such as the feeding, housing, transportation and storage of specific insect species and their subsequent food/feed products. This research will prevent current environmentally taxing food production methods being replaced with equally harmful insect production methods. Ultimately, insects are not a panacea, but may be a step in the right direction towards providing nutritional food for an exponentially increasing global population, whilst reducing the environmental footprint of traditional agricultural systems.

Research and commercial activities into insects as viable sources of protein for feed and food products have accelerated over the past decade, in line with increased consumer demand for environmentally friendly food. However, studies have reported challenges to the scaling up insect production systems, with many gaps in knowledge, all requiring careful analysis before the environmental benefits of edible insect products can be quantified^5^. The sustained growth of the edible insect industry faces many challenges, especially in Western countries from consumer barriers, restrictive novel food legislation, concerns in emerging food allergy, the safety of insect processing techniques and possible fraudulent supply chains^14,15^, all which need addressing before any significant shift towards entomophagy in Western diets.

The rearing of insects as novel safe and sustainable food products has shown to give both benefits and challenges. Some of the limitations hindering the global reach of the edible insect’s industry have been highlighted such as a lack of production legislation, and the challenge of the use of waste streams as feed substrates to reduce the ecological impact of production.

### The main aims of this review and how the analysis was conducted

With a growing curiosity and demand among Western consumers, insect proteins remain at a high risk of food fraud and their vulnerability has not yet been reviewed. The main aim herein is to map out the insect supply chain, based on current publications and online interviews with key players within the edible insect industry in the European Union. Due to a lack of evidence on fraudulent activity within the edible insect industries, this study has analysed all available literature, EU legislation on food fraud and databases regarding edible insect proteins. This has identified points of fraud vulnerability along the supply chain so that potential mitigation measures could be suggested to combat potential frauds.

Three approaches namely (1) a literature search, (2) a systematic investigation of food fraud databases and (3) online interviews with key stakeholders within the edible insect industry were conducted to map out the insect protein supply chain and to identify points where safety hazards and fraud activities are most likely to occur.

#### The literature search

Online peer-reviewed literature databases, Web of Science Core Collection and SCOPUS, were searched for relevant literature on the production of edible insects, including processing and the potential food safety risks. The databases were searched using terms specific for this review: in general, ‘edible insect’ OR ‘entomophagy’, for food safety, ‘food safety’ OR ‘microbiological contamination’ OR ‘chemical contamination’ OR ‘supply chain’ OR ‘processing’ and for food fraud, ‘food fraud’ OR ‘fraudulent activity’ OR ‘authenticity’. Due to the limited previous research on the safety aspects of entomophagy, the search was not narrowed by publication year, country, or other parameters. Selection was employed to identify articles relevant through their title. After this test, the abstracts were screened, and for those deemed suitable, the full papers were examined. Some papers were excluded if they were beyond the scope of this review, looking at consumerism barriers towards edible insects in Western countries or the cultural importance of entomophagy in Eastern countries. In addition to online databases, a grey literature search was conducted to identify relevant articles and documents from governmental agencies on the potential integration of edible insects into Western European diets. The grey literature used in this review was primarily sourced from European governmental agencies such as the EU Commission, and European Food Safety Authority (EFSA). From these, additional authorities such as the World Health Organisation (WHO) and Food and Agriculture Organisation (FAO) were sourced to provide information regarding the design of laws and regulations of the production, trade and consumption of edible insects on the EU market. Literature which identified evidence for hazards associated with insects consumed as food was systematically assessed from late 2021 to early 2023.

#### The systematic investigation of food fraud databases

Rapid Alert System for Food and Feed (RASFF), an online food safety system and Tridge, the on-line food and agriculture database network for trading commodities were found through a Google search of ‘EU food fraud databases.’ They were used to identify food safety and authenticity issues of edible insect products using the key terms, ‘insect protein’ or ‘edible insect’. The notifications (RASFF, 23 and Tridge, 2) were assessed based on their relevancy, and subsequently included or excluded from this review as most of the results sourced food safety notices of insects being a contamination in food, rather than identifying it as the food itself which is not within the scope of this review.

#### The online interviews with key stakeholders

A total of 18 individuals from food companies and entomophagy researchers were contacted, of these only four individuals from companies, and one researcher replied. Of the four industry stakeholders, two were from European insect food-based companies, (one involved in the rearing and processing, and the other solely processing), the third was from a European insect feed company (involved in processing only), and the last was an individual from a Northern Irish meat company with interest in expanding to the insect proteins market. The academic individual is a Senior Lecturer at Queens University, Belfast with a research focus on alternative feed sources for livestock, including insects, with a goal of identifying technologies and nutritional practices to reduce the environmental impact of conventional sources of protein.

The interviews were carried out in three steps. Firstly, the content and main aims of this research were explained. Secondly, the interviewees were asked to introduce themselves, their companies and their roles within the insect industry. Lastly, the interviewees were asked to answer questions about their products, supply chains and potential areas of food safety and fraudulent activities, the questions used are in Supplementary Data Section. The information from interviewees and their affiliated companies was confidential and not to be published. The information obtained was held in line with General Data Protection Rights and used in mapping out the insect supply chain. The interviewee’s opinions about food safety and fraudulent activities around insect proteins and products helped to locate the potential vulnerabilities along the insect protein supply chain.

### The approval and expansion of insect species onto the European market

With growing interest towards edible insects in the EU, owing to their potential environmental and food security benefits, EFSA^16^ published their first scientific risk assessment on food safety and allergenic concerns associated with the production and consumption of insect products intended for feed and food. According to the EU’s regulation on Novel Foods (NF), which encompasses edible insects (EU 2015/2283), food companies who wish to place NF’s on the EU market must submit a company-specific application for authorisation. When accepted, this grants the company the sole right for marketing this product for 5 years, and a safety assessment by EFSA is published within 9 months of verified application^16^. It may be the case that multiple companies hold authorisation of the same insect species as a novel food product, such as *Tenebrio molitor*, however, when applying for authorisation it is likely that food companies will request data protection of their product and methods. The most recent EU regulations (2017/2469 and 2017/893) specify stringent requirements of insect protein production regarding substrate used when applying for the commercialisation of insect products onto the market^17^. It could be argued that these restrictive parameters hinder the potentially exponential growth of the EU insect market, however, these regulations are developed under the Precautionary Principle to ensure food and feed safety of a largely under researched industry.

Among the over 2000 known edible insect species, only four, namely yellow mealworm (*Tenebrio molitor)*, migratory locust/grasshopper *(Locusta migratoria)*, grain mould beetle, also known as lesser mealworm which is a larval form of *Alphitobius diaperinus* (from the family of Tenebrionidae of darkling beetles) and house cricket *(Acheta domesticus)*, are currently authorised in specific products through specific producers in the EU^18^. For example, SAS EAP Group, France submitted the request for dried *Tenebrio molitor* larvae to be used as whole, dried insects in snacks and as a food ingredient^19^. In addition to this, Nutri’Earth and Ynsect in France and the Belgian Insect Industry Federation in Belgium submitted NF applications for *Tenebrio molitor* within their products. Following a stringent scientific and risk assessment by EFSA, these insects have been approved within these specific products. Currently, eight novel food applications for insects are awaiting safety evaluations to include *Alphitobius diaperinus* by Proti-Farm Holding NV in the Netherlands and *Hermetia illucens* by Enorm Biofactory in Denmark^18,20^. These applications and authorisations are significant milestones in the acceptance and expansion of the edible insect industry in Europe, as consumerism barriers intrinsic to this type of food product are challenged and broken down.

Due to the demand for sustainable, high protein foods over the past decade, market evaluations by the International Platform of Insects for Food and Feed (IPIFF)^21^ have forecast that 39 million EU consumers will incorporate insects in their diet by 2030, an increase of over 400% from 9 million in 2019. However, this expansion poses challenges for EU regulatory food safety bodies in the safety processing methods and the viability of utilising organic waste streams as insect feed. Continued research and risk assessment into entomophagy in Western countries will support the approval of insect products in the EU and encourage our food supply to become more regenerative and circular to provide for an exponentially increasing global population.

### Different forms of insects for consumption and their by-products for other applications

The global edible insect market, as a niche but expanding industry among Western countries, is not only segmented by the geography of consumption, insect species, the forms in which they are consumed and the agricultural uses of the organic waste they produce). Whole insects, whether fresh or processed (roasting, frying, or boiling), are consumed mainly in Eastern countries such as Thailand and India, where entomophagy is part of their traditional cultures, whereas edible insects in Western countries are often fortified into familiar food products such as baked goods, pasta or snacks as ground or powdered forms^7,22^. Of 500 tonnes of insect-based foods produced by EU businesses in 2019, approximately 75% was in the form of powdered insect ingredients^21^. Incorporating ground insects into familiar foods helps to break down negative consumer barriers of disgust and aversion to entomophagy among European consumers. However, this processing may expose EU insect products and snacks to fraudulent activity along the supply chain.

Before processing into a powdered protein, the fats and oils of edible insects can be extracted usually by method of mechanical pressing or aqueous based oil extraction. More than 30% of the total weight of lesser mealworm is fat content^23^, and although species-dependent, these oils contain easily digestible fatty acids and other essential nutrients, making it an excellent high energy animal feed product^24^. However, whether fats and oils are extracted prior to processing is dependent on the intentions of the final product, such as its desired nutritional profile, texture and use within the food industry. Furthermore, the waste stream produced from insect production, consisting of moulting skin (exuviae) and insect faeces, is collectively known as insect frass^25^, has been reported to be a high-quality soil fertiliser due to its high nitrogen, potassium, and phosphorus content (see Table 1)^26–30^.

**Table 1:** The common forms of insect’s products and their organic waste.

Form	Description of insect product/waste	Visual
Fresh whole insects	Fresh whole insects are sold on the market with no processing. These products are usually found at street food markets in Eastern countries such as China and Thailand^7^.	^26^
Whole dried insects	Whole dried insects are whole insects which have had their moisture removed by drying. Drying may be simple air drying or by means of heat^27^. These techniques are popular in countries where entomophagy has been practiced for years such as Asian countries. There is very little consumption of whole dried insects in the EU at current, without further processing.	
Ground dried insects	Ground dried or powdered insects are obtained by drying insects, usually through heat or freeze drying. For some species such as locusts, the head and legs are removed. Next, the remaining insect is ground into a fine powder^7^. This form is gaining popularity in Western countries to fortify familiar foods and to increase the protein content such as protein bars and baked goods.	
Insect oils/fats	Insect oils are the fats which have been extracted from insects, usually by means of fractionation, mechanical pressing or aqueous based extraction methods. Currently, the main application of insect oils is in the animal feed industry as a high energy, high fatty acid additive, however, some studies have shown potential applications as food ingredients or table oils^28^.	Insect oils: Beetle Larvae (LM), Cockroach (CO) and Cricket (CR)^29^
Insect frass	Insect frass is a by-product obtained from insect food and feed production to include spent feedstock, faeces and exuviae^25,30^. Therefore, insect frass contains chitin from the exoskeletons of insects, which is broken down by microorganisms in the soil. High in nitrogen, potassium and phosphorus, insect frass can be used to improve the quality and fertility of soils.	^26^

Pictures for whole dried insects and ground dried insects were captured by the author.

### The edible insect supply chain

The supply chain and trading channels of insects depend on their geographic origin and country of intended consumption. In Asian countries, such as Thailand, the insect supply chain is generally short with a small-scale distribution as they are minimally processed or packaged^31^. However, the edible insect supply chain in EU is more complex, given the need for processing into other food products due to lingering consumerism barriers^32,33^. Pippinato^34^ reported 65% of EU insect company’s imported whole insects from Asian countries to process and retail in the EU, while only 12 out of 59 EU insect companies produced their own raw materials.

**Fig. 1:**
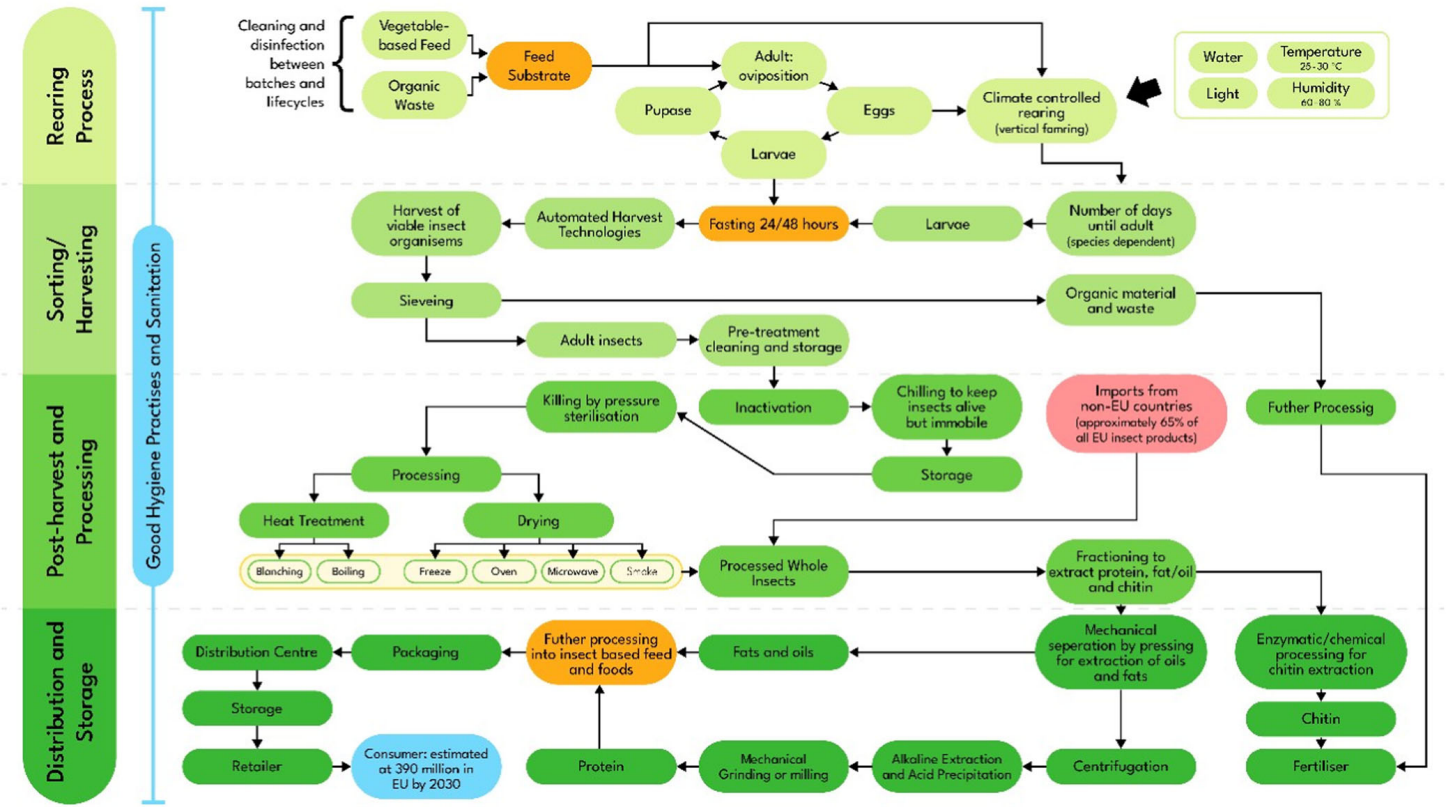
The Edible Insect Supply Chain, representative of EU insects and products. This figure has been mapped out with aid from online interviews with key stakeholders along the insect supply chain and analysis of scientific studies on the rearing and processing of insects, and EU governmental risk assessments detailing food safety parameters during the production of edible insects.

The EU edible insect supply chain follows a traditional food commodity including the rearing, harvesting, and processing (Fig. 1). This figure has been mapped out with aid from online interviews with key stakeholders along the insect supply chain to include individuals from EU insect food and feed companies, and an individual involved in academia focused on the viability and sustainability of insects as a feed source. In addition to this, the remaining parts of the supply chain were mapped out through analysis of scientific studies on the rearing and processing of insects, and EU governmental risk assessments detailing food safety parameters during the production of edible insects (Table 2^35–44^).

**Table 2:** Review table to summarise the literature used to map the EU edible insect supply chain.

Author, year	Insect Species	Step in supply chain	Study description and process
IPIFF^21^	Applicable to all insect species	Feed substrate	Assessment of EU legislation on edible insects as ‘farmed animals’ and the prohibition of organic food waste streams as insect feed substrate
Fowles & Nansen^35^	Black soldier fly, housefly, yellow mealworm, Cambodian field crickets	Feed substrate	Exploration of suitable food-waste-to-insect species pairings to increase the capacity, efficiency and safety of using food waste as feed substrate
Ojha et al.^36^	Applicable to all insect species	Feed substrate (circular economy of insects)	Assessment of the current status of insect processing and their potential contribution to a circular economy through food waste valorisation
Wynants et al.^37^	Mealworm larvae	Starvation	Mealworm larvae were starved 24 and 48 h before harvest. Starvation did not reduce microbial numbers when compared to control group
Inacio, et al.^38^	House cricket	Starvation	24 and 48 h starvation before harvest increased the Total Aerobic Count (TAC) and decreased fat content of insects
EFSA^22^	Applicable to all insect species	Feed substrate, harvest and processing	Scientific risk profile to assess the potential biological, chemical and allergenic hazards associated with edible insects in feed and food. Through this, the entire supply chain was taken into account
Caparros Medigo et al.^39^	Fresh mealworms, house crickets, smoked termites and caterpillars	Processing (blanching, freeze-drying and sterilisation)	Processed samples had a lower TAC than untreated insect products, portraying that processing improved the food safety of edible insects, however, the efficacy of processing treatments was often species dependent.
Klunder et al.^40^	Mealworm larvae and house cricket	Processing and storage	Assessment of the microbial load of fresh, processed and stored mealworm larvae and house cricket. A short heating step was effective in eliminating *Entereobacteriaceae*, but some spore-forming bacteria remained. Drying and acidifying methods were promising, but on a species-to-species basis.
Melgar-Lalanne et al.^41^	Applicable to all insect species	Traditional and novel processing techniques	Highlights the range of different processing techniques of edible insects, both conventional and novel, but also the lack of research into the efficiency of these specific to insect species.
Amarender et al.^42^	Crickets	Protein and lipid extraction	A combination of lipid extraction using ethanol and protein extraction by method of using sodium hydroxide or ascorbic acid to maximise the efficiency of protein extraction.
Luo et al.^43^	Cicada slough, silkworm chrysalis, yellow mealworm, grasshopper and shrimp shells	Chitin extraction	A comparison of the physiochemical and morphological characteristics of the chitin exoskeleton of insects through alcohol based extraction methods
Soon et al.^44^	Superworm (*Zophobas morio)*	Chitin extraction	Deproteinisation of protein content in chitin of superworms through method of sodium hydroxide extraction
Tzompa-Sosa et al.^28^	Yellow mealworm, lesser mealworm, house cricket and the Dubia cockroach	Lipid extraction	Comparison of aqueous-based lipid extraction methods and organic solvent-based extraction methods, and the effect this had on fatty acid composition per insect species.

Only adult insects have been mapped as one of the final products, not their larval form, as the review analysis into processing and its subsequent effects on food safety have been investigated on adult insects. In addition to this, organic waste has been included as a feed source for insects within Fig. 1 to highlight the potential self-sustaining, environmental benefits of edible insects through their circular economy. However, as an emerging industry with increasing demand for sustainable food within Western countries, EU insect industry has to broaden their sourcing globally to non-EU insect producers. The supply chain map (Fig. 1) with substantial import and export avenues along the insect supply chain in EU shows particular points of vulnerability in safety and fraud within the supply chain.

From the insect supply chain (Fig. 1), feed substrates can be seen as sources of microbiological and chemical threats to the supply chain, through harmful bacteria, heavy metals, viruses, mycotoxins and prions^45^. However, the environmental benefits of insects in feeds and foods are based on the nutritional flexibility and ability of insect species to utilise a range of food waste streams as feeds, converting them into high quality proteins^35,46^. This reduces the need for resources such as water and arable land during food production, whilst also reducing the need for feed from other livestock industries. According to UNEP^47^, almost 20% of global food available to consumers was wasted in 2019 and some would argue that this could have been used as a feed substrate for insect protein production. However, there is much concern for the safety of subsequent food and feed chains from waste streams as insect feed. Waste food may contain foodborne pathogens and chemical hazards, harmful to human health^36^, and therefore, under EU food and feed laws (Regulation 1069/2009), the use of waste stream feeds for edible insect production is prohibited^21^.

EU legislation does not provide ‘insect-specific’ guidance on microbiological or chemical limits in substrates, but these must meet the same regulatory and contamination guidelines as animal feed, according to Directive 2002/32/EC^48^. Therefore, the safety of subsequent insect feed and food chains is largely dependent on the rearing conditions and feed substrate of insects. Data from IPIFF^21^ has revealed that only 28% of EU insect feed and food companies are involved in the rearing, right through to processing the final product, leaving most companies to source whole dried insects to further process into products fit for EU consumers. Consequently, the regulation and monitoring of feed safety parameters during the rearing of insects intended for the EU feed and food markets is paramount to ensure the safety of these supply chains and will prevent any hazardous materials impacting negatively upon human and animal health.

Numerous studies have been carried out to investigate the viability of food waste streams as feeds across a range of insect species. A plethora of waste streams has been explored, including spent grains, beer yeast, potato peelings, waste plant tissues and grocery store food waste after aerobic enzymatic digestion^49–51^. Some of these sources require additional processing to ensure their microbiological safety before being used as insect feeds, but few economic evaluations exist on this aspect^35,52^.

In the race to provide an increasing world population with safe and alternative proteins, there has been much investigation into the differing levels of bioaccumulation of heavy metals and other contaminants among edible insects through feed substrates. Camenzuli^53^ conducted feeding trials spiked with different mycotoxins (Aflatoxin B_1_, deoxynivalenol (DON), ochratoxin A and zearalenone) at levels above those of the EU’s maximum limits and reported no harmful accumulation in the larvae of black soldier fly or lesser mealworm. Van der Fels-Klerx^54^ investigated the viability of *Tenebrio molitor* (yellow mealworm) and *Hermetia illucens* (black soldier fly, BSF) larvae as a safe food source by assessing bioaccumulation of cadmium, lead, and arsenic in spiked feeding trials whereby insect feed substrate was spiked at 0.5, 1, and 2 times those of EU maximum allowable limits (ML). The lead and arsenic concentrations in BSF residual material were higher than in the BSF larvae, conveying that these species were efficient in excreting these two elements. However, in the mealworm, arsenic concentrations were lower in residual material than the larvae, whereas lead concentrations in the mealworm residual matter were up to 60 times found in the mealworms. Dallinger^55^ studied the ability of insects to inactivate toxic metals through intracellular compartmentalisation by method of vesicle sequestration of toxic substances into their exoskeleton, and it could be suggested that higher lead concentrations in the BSF larval exuviate than in larvae or feed is due to sequestration into the insects’ exoskeleton. Other research has been conducted in this area, Lindqvist^56^ and Pederson^57^ have concluded that BSF larvae have a significantly higher number of Ca^2+^ channels compared to other insect species, and from this they have theorised these channels to facilitate removal of toxic substances. When a high heavy metal concentration is detected, these channels actively pump heavy metals across the cell membranes, and into vesicles which inactivate and store the toxic substances within the insect exoskeleton. The heavy metals no longer threaten the insect’s health, and this would therefore explain the accumulation of cadmium in the exuviate of these species. However, this area of investigation is under researched and requires further investigation from the scientific community. Consequently, it can be concluded that individual element uptake and bioaccumulation of harmful compounds in insects varies from species to species, including their ability to excrete heavy metals through faecal matter or inactivation through vesicle sequestration, calling for further research and revision into the EU’s ML for toxic substances in insects.

The additional processing and transport of insect products intended for consumption in Western countries add to the carbon footprint of production^58^. These additional processing and transport are energy intensive, as modelled by the life cycle assessment of black soldier flies by^59^. The question to be asked is how much more sustainable, if at all, is edible insect production when compared to other conventional sources of protein? Despite this, many studies have shown edible insects to have much lower ecological and carbon footprints than conventional sources of protein such as beef and poultry, with the majority of energy usage in insect production used for the maintenance of climate-controlled conditions for the poikilothermic species^60–62^.

Baiano^63^ has predicted the EU and North American edible insect market to grow by up to 43% by 2024. Therefore, for insects to be considered a safe and viable protein source, it is crucial that EU ML of microbiological and chemical contaminants (Reg 2017/2470)^16^ are continually assessed on a species-to-species basis as insect products continued to be approved onto the EU market. The insect industry boasts their potential sustainability advantages over conventional sources of protein production; however, this is dependent on further investigation into the safe utilisation of waste streams as feeds. Consequentially, the long-term sustainable insect production in Western countries lies with specific legislation to guide the use of safe, economic, and high-quality waste streams as insect feed substrates.

### The microbiological, chemical and allergenic threat of edible insects towards human health, and potential mitigation measures to improve food safety

An extensive risk assessment by EFSA in 2018 among other scientific studies has identified high bacterial numbers within edible insect production and alarming food safety concerns^63^. Some of the studies identifying the microbiological threats found in edible insects include *Salmonella spp*. and *Enterobacteriaceae* in black soldier fly^64^; *Bacillus cereus* in black soldier fly^65^ and yeasts and moulds in house cricket^66^ to name a few.

Klunder^40^ reported findings of *Enterobacteriaceae, Lactic Acid* Bacteria *and* Bacterial endospores present in a study investigating the microbiological content of whole edible insects as fresh, processed and stored. He reported increased bacteria present in edible insects, after implementing a ‘crushing’ step before processing and consumption. The Total Viable Count (TVC) in lesser mealworm increased from 1.7 cfu/g in whole larvae to 2.5 cfu/g in crushed larvae, even after 10 min of boiling. Similarly, whole roasted lesser mealworm had 2.2cfu/g of *Enterobacteriaceae*, and this increased to 2.6 cfu/g for larvae that has been crushed, even after 10 min of roasting. Klunder^40^ hypothesised that this was likely the result of the release of bacteria-containing waste from the insects. This has been the only scientific study in literature which carries out a ‘crushing’ step, and consequentially, the microbiological threat to subsequent food chains has increased. However, some producers have recognised this and implemented a 24–48 h ‘fasting’ or ‘starvation’ step before harvest as a prerequisite for safety in subsequent feed and food chains. The purpose of this is to eliminate any potential microbiological and chemical hazards consumed by insects in their feed and passed onto human food chains^67^. However, most studies have highlighted the inefficiencies of this pre-harvest safety method. For instance, Wynants^37^ reported that a 48-h starvation period did not lower the gut microbiota in edible insects. Similarly, Inacio^38^ reported an increase in the total aerobic count (TAC) from 7.3 log cfu/g in the control group to 7.8 log cfu/g and 8.2 log cfu/g for the 24- and 48-h starvation groups, respectively. Furthermore, Inacio^38^ reported starvation decreased the fat content in insects, with potential profit losses for producers and nutritional benefits for consumers. Thus, these studies indicate that starvation is an ineffective means to reduce microbial loads in edible insects. Moreover, controversy has arisen on the ethics of this practise, as insects are classed as ‘farmed animals’ under EU legislation^16^. However, due to associations as pests and diseases, most Western consumers do not view insects as animals, and in the interests of safety, do not see moral implications of this practise.

In addition to the potential microbiological and chemical threats of edible insects, it has been discovered that a subpopulation of allergic individuals are more susceptible to risk of edible insects due to allergenicity concerns sources^45,68^. In a recent FAO review^69,70^ growing trends in food allergy driven by alternative proteins, including insects, have been reported. Although very few direct reports of clinical incidents or research on how insect proteins may have led to anaphylaxis can be found currently, the similarity of tropomyosin epitopes (as one of the most commonly known allergens found in seafood) among Arthropods may help foster an understanding of the allergenic mechanisms. Therefore, humans which are allergic to shellfish/crustaceans and dust mites are likely to experience sensitisation and subsequent allergic reactions if insect proteins are consumed, and this is attributable to cross-reactivities among the protein structures^71^. IgE-mediated cross-reactivities with shrimp have been reported for crickets (Acheta domesticus)^27^ and orthopteran (Gryllus bimaculatus)^72^. Meanwhile, insect proteins have displayed a diverse range of TM-associated IgE reactivity^73,74^ which were linked to its amino acid sequence^75^ and structure^76^. Identification of novel allergens in edible insect have also been reported^77^ and in Gryllus bimaculatus together with cross-reactivity to Macrobrachium spp^78^.

Scientific studies investigating the risk from allergens and their detection have concluded that the potency of allergens found in differing insect proteins respond under different processing techniques^7,27,79,80^. Pali-Schöll^81^ determined that when exposed to severe heat treatments or enzymatic hydrolysis, the immunoreactivity of migratory locust was eliminated, whereas studies by De Marchi^27^ and Leni^75^ highlighted the inefficiencies of such treatments on the immunoreactivity of insect proteins found in house crickets and black soldier fly. In addition to this, it is important to consider the impact of the food matrix on the allergenic potency of insect proteins, as due to consumerism barriers posed by Western consumers, insect proteins are often added to familiar food products such as pasta or bread to enrich their nutrient qualities^82^. Other evidence found from dogs has revealed that insect protein may also pose allergy risks in animals as feed^83^. However, more studies and a larger evidence base are needed to enhance our current knowledge about food allergy risks that will be introduced by insects.

From these studies, it is apparent that further research into the allergenicity of specific insect proteins is required to determine the route of sensitisation, and the minor and major allergens associated with each of the four insect species approved as a novel food by the EU. As more insect-based foods trickle into the EU food market, it is imperative that the effect of processing on the safety of insect-based foods needs further investigation to provide a representative risk profile of the consumption of insect proteins on human health, to include microbiological, chemical, and allergenic hazards.

### The effect of different processing methods on differing insect species regarding the food safety of the final product

The processing of insects has also been identified as a vulnerability within the edible insect supply chain (Fig. 1). Unlike in Eastern countries where insects are often minimally processed to improve their sensory qualities and shelf life^41^. In Western countries, insects undergo more intensive processing through methods such as dry fractionation and enzymatic degradation into proteins, fats and chitin for various applications in the feed and food industries^41^. The extensive processing is required to ‘mask’ insects within familiar food products due to Western consumers’ view of insects as pests and disease vectors, rather than as sustainable and nutritious protein sources. These processing steps improve their food safety as heat treatment or drying methods reduce total counts of microbes^40,84^. However, it is important to consider the microbial content of insects before harvest as Vandeweyer^66^ reported stable water activity (a_w_) (0.35 ± 0.04 and 0.98 ± 0.01) during the storage of oven-dried crickets and frozen crickets, respectively. However, smoked crickets displayed a marginal increase in a_w_, showing the importance of appropriate processing and preservation of products, prior to use. Furthermore, Vandeweyer^85^ confirmed that microbial load of edible insects remained stable for up to 6 months in differing processed products, presenting insect products as a safe, sustainable source of protein for health and environment-conscious consumers. Despite these studies, gaps remain about the safety of processing techniques and the microbiological content of finished insect products, and therefore the need for further extensive research is evident.

Moreover, according to the IPIFF^21^, 36% of insect-based food companies in the EU are involved in the final processing of the insects for food, with less than a third involved in all stages of production, from rearing to the final product. Thus, most of the insects in EU insect snacks are imported, in agreement with findings of Pippinato^34^. However, this processing, to meet Western consumer demands, coupled with import avenues of whole insects to be processed and sold in the EU, lengthens the supply chain, exposing it to a greater risk of safety and fraud concerns. Thus far, the risks of food fraud within these alternative protein products have not been systematically studied.

### The fraudulent concerns in insect proteins

Food fraud is a global issue of mounting significance, with the potential to cause harm to human and animal health^86^. Although the globalisation of our food chains brings many benefits to food choices and supplies, it can expose global food supply chains to fraudulent dealings. Similar to all food and commodities, the motives of committing insect protein fraud are economically driven, with low chances of being detected at the present time.

Although there is no globally agreed definition of food fraud as yet, it can be characterised by the ‘illegal intentional deception for economic gain using food’^87,88^ and this can take form in many ways such as the deliberate adulteration of foods through addition, dilution, replacement or falsifying ‘country of origin’ or production systems. As the complexity of food supply chains grow, the transparency and control of the supply chain weakens. The mapping of the insect supply chain in this study illustrates the complexity that exists.

### Sparseness of research into edible insect fraud

As a novel and largely under-researched food product, risk assessment into the vulnerability of insect products to adulteration is almost non-existent. When “‘insects’ AND ‘fraud’” were searched on Web of Science and SCOPUS, a total of 17 articles were found^89,90^. Of these, only two articles investigated the potential of fraud within insect products in relation to feed, while others primarily explore insects as being an undeclared and unauthorised pests present in foods, rather than as a type of food product. No published study was found which investigated the potential fraud in edible insect food supply chains. This is the first study, to the author’s knowledge, to begin to address this topic. Insect-specific food authenticity regulations and standards are non-existent, which greatly hampers combatting illegal activities along supply chains^91^.

**Fig. 2:**
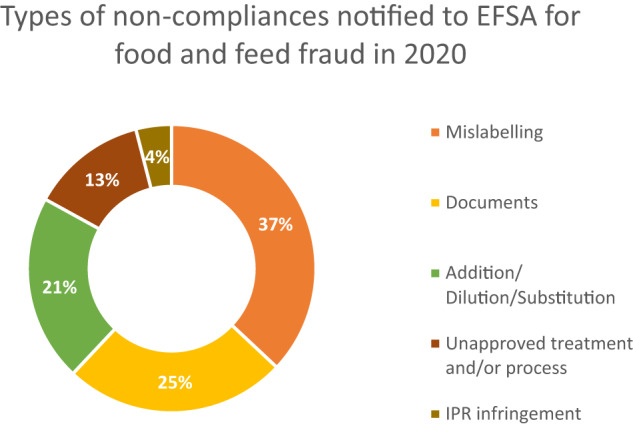
The relative proportion of the types of non-compliance fraudulent activity within food and feed products notified to EFSA in 2020.

Among various types of fraudulent non-compliances reported to EFSA in 2020 relating to food or feed, mislabelling and documents represent almost two thirds of fraudulent activity reported (Fig. 2). Furthermore, Table 3 describes the different possible types of fraudulent activity in the food insect industry.

**Table 3:** The different categories of fraud as notified to EFSA with the potential application of each type of fraud in the edible insect industry.

Food/feed fraud:	Description of Fraudulent activity	Potential application to the edible insect industry
Mislabelling/ Documents	Fraudulently placing false or misleading claims or information on product packaging, often to appear as a premium product and thereby increase profit margins. Mislabelling fraud may encompass false nutritional, geographical, expiration date or quality claims.	False mislabelling of country of origin, production methods or nutritional information such as protein or fats content, etc. to deceive customers into thinking this is a premium product, thereby increasing the products price and profit margins
Documents	The process of tampering, adapting or imitating documents relating to the identification, origin or production of food products such as animal passports and identification.	Documents relating to insect species, production methods, or feed substrate could be tampered with.
Addition	The process of intentionally adding undeclared substances or elements into food/feed products which have not been approved and are not declared on packaging. The substances added are often of an inferior quality for the purpose of ‘bulking’ the product for financial gain.	Undeclared substances which boost nitrogen content such as melamine could be added to insect products. The illegal addition of melamine or other nitrogenous compounds would artificially inflate the apparent insect protein content upon analysis. A major food safety risk would result.
Dilution/ Substitution	The process of intentionally diluting or substituting a product of high value within a food product with another nutrient, ingredient or food, often of a lower value for financial gain. Dilution often refers to liquids, whereas substitution refers to food products.	Insect protein powders could be substituted with lower quality substances such as sawdust to increase profit margins. Other forms of lower cost protein (soya for example) could be used as adulterants and thus trigger food allergy issues.
Unapproved treatment or process	The process of intentionally carrying out unapproved treatments during food production, and this can refer to the use of pesticides, chemicals, veterinary medicines and growth promoters during production, or the incorrect handling, packaging, transport and storage or products.	The use of illegal or undeclared chemicals during insect production such as pesticides or veterinary medicines which have not been declared on the packaging.
Concealment	Fraudulently hiding the quality of ingredients or products such as contaminated, putrid meat and fish products treated with unauthorised food improvement agents or additives.	Deceiving consumer through incorrect and non-transparent quality of the product and nutritional information such as the protein, fats, vitamin or mineral content.
IPR infringement	Intellectual Property Rights (IPR) infringement refers to the fraudulent replication of any aspect of genuine packaging such as copying the brand name, logo, packaging or processing method for economic gain.	As an emerging industry in Western countries, with few companies in the supply chain, it could be the case that unauthorised products are fraudulently labelled with reputable supplier’s names and logos i.e., counterfeiting of bona fide products

### The Melamine Scandal as a possible model for fraud in the edible insect industry

As a largely under examined sector, there are a wealth of opportunities and vulnerabilities within the edible insect industry for fraudsters to exploit as indicated in Table 3. For years, it has been known that the increasing demand for protein-containing foods has placed them at increased risks of adulteration^92^. For example, in the 2008 Melamine Scandal in China, where dairy products and infant formula were adulterated to boost their nitrogen content, giving the illusion of higher protein content and thus higher value^87,93^. This nitrogen rich compound could not be distinguished from genuine milk proteins using the industry standard Kjeldahl^94^ and Dumas methods of analysis. Their protein content was artificially inflated for financial gain and was sold into the market without any thought or understanding for the potential food safety crisis that would ensue^95^. Melamine, used in the production of industrial glues and plastics, was and is not approved for use in foods and feeds due to its toxicity^96^. Its addition to milk, infant formula and other milk derived products in China resulted in six infant deaths and over 300,000 illnesses^87^. The present authors have concerns that one of the most serious forms of fraud that may be perpetrated in the insect protein industry could be modelled on the Melamine Scandal.

For approval of a novel food in the EU market, the product or ingredient must undergo risk and food safety assessments for the protection of public health by EFSA. This also includes an investigation of the products nutritional value, and this data analysis of these methods has highlighted potential over-estimation of the protein content in edible insects. Through the Kjeldahl method of protein content measurement with a nitrogen to protein conversion factor of 6.25^94^, the EFSA’s risk assessment for the market approval of house cricket and yellow mealworm reported an ‘overestimated protein content’^22^. It was concluded that the inaccuracy of protein content was due to the detection of non-protein nitrogen from the exoskeleton of insects^22^. However, this is of concern for the transparency and authenticity of edible insect products. This ‘loop-hole’ in analytical method as a result of the body composition of ground insects by the Kjeldahl approach presents an opportunity of fraud with these products.

Similar to the outworking’s of the Melamine Scandal, it is likely that fraudsters will exploit this similar vulnerability for financial gain. Many would argue that the Melamine Scandal was due to the uncontrolled expansion of the dairy industry in China, through demands of an exponentially increasing population. This could be used as a prediction model for potential of fraudulent activity within the recent growth of insect-based industries, which has been forecast that 390 million EU consumers will eat insects in 2030, a four-fold increase from the 2019 figure^22^. This raises the question as to whether the edible insect supply chain is robust enough to sustain the increased production of safe and authentic products in line with the increased demand. A thorough understanding of the Melamine Scandal as a predictor model might allow mitigation strategies for fraud with edible insects to be developed. This calls for the urgent implementation of regulatory testing and guidance for food businesses regarding novel protein foods for the prevention of fraud and retention of consumer trust.

Most food fraud frauds are motivated by financial gain, and this will not be an exception in the edible insect industry. On average, cricket protein powder costs £48.75/kg (Eat Grub; Gymsect; Bugvita; Cricket Hop Co.), which is more expensive when compared to other protein sources, such as vegan blends (£29.99/kg), soy protein isolate (£22.99/kg), pea isolate (£17.99/kg) and impact whey (£37.99/kg) from MyProtein^97^. Consequently, the higher prices of insect proteins compared to other protein sources is another factor which places them at greater risk of fraud. With a premium price among protein products coupled with an increasing consumer demand in Western countries, fraudsters may recognise this opportunity to exploit this novel industry and profit through fraud. However, with continued approvals of insect species onto the EU market, coupled with an increased consumer demand, the upscaling of production and trade in the EU is expected to bring costs down. In addition to this, the stringent application process, including detailed supplier regulations and processing parameters involved with authorising edible insect products onto the EU food market may act as protection against food fraud. Only company-specific applications which have been approved by the EU Commission as a novel food product can be legally sold on the EU market thereby acting as a deterrent for exploitation by food fraudsters.

The approval of edible insect species onto the EU market as novel foods, coupled with an increased consumer demand for sustainable protein sources will allow these foods to move from niche to mainstream markets. Outdated attitudes regarding insects as pest species are changing as younger, health-conscious generations have become increasingly accepting and inquisitive about edible insects due to their nutritional and sustainability benefits. However, many gaps remain in the production and safety of these novel foods, such as a lack of insect-specific legislation in regard to maximum contamination limits of microbiological and noxious chemicals in products. This lack of safety assessment hinders the market expansion of edible insect containing foods. This first mapping of the edible insect supply chain in this review will, in the view of the authors, be instrumental in identifying points of vulnerability in terms of food safety along the supply chain to ensure consumer protection.

Food fraud is a constant threat in all regions of the world, particularly to new supply chains such as edible insects. The aversion of edible insects from Western consumers may expose these novel foods products to food fraud as insects are often ground into a fine powder and incorporated into other more familiar foods. This makes these novel products more appealing to consume within the EU market, but consequently, an easy target for food adulteration for financial gain. Therefore, it is challenging to identify and quantify the fraud risks within the sector. This study has used information gained from interviews with key stakeholders along the insect supply chains, what is available in the literature and the Melamine Scandal as a model to identify potential supply chain frauds.

Equally, as an increasing number of insect novel products enter our supply chains and supermarkets, consumers may not be aware of the potential dangers associated with the consumption of edible insects. Therefore, as the popularity of insects in the Western diet continues to climb, it is imperative that we protect the insect industry from harmful and reputation-damaging fraud, whilst protecting consumers of the potential food safety and allergenic risks associated with insects. Similar to all food products, it could be argued that in one case, edible insects may be the target of fraudulent activities, whereas in other situations, insects are the means of harm to humans and animals. Therefore, the insect supply chain mapped out within this review will help key stakeholders to identify and establish precautionary measures towards areas of vulnerability of this novel industry towards fraud. For example, protein analysis techniques used on insect powders and products should not use the Kjeldahl method of analysis to avoid the over-estimation of protein content and prevent an opportunity of exploitation of these products to arise for fraudsters to avail of at the expense of consumer health. Similarly, the EU’s stringent guidelines and regulations of legally authorising an insect product onto the market will protect the industry and consumers alike and allow edible insects to have a positive impact within our supply chains, on human health and the sustainability of food production methods for an increasing global population.

To conclude, insects have come a long way from being seen as purely pests, to food in niche markets and to industrial production for the European consumer market and beyond. However, much research and assessment are still required to fully understand the future safety and fraud risks of insect-based foods. Without this information consumers will be at risk and the burgeoning market may suffer from a serious loss in confidence if a safety and/or fraud major scandal occurs.

### Supplementary information

Supplementary Material

## Data Availability

We declare that data sharing does not apply to our manuscript. This is a review article with no data analysed during the study.
