# Supplementary material for: An analysis of emerging food safety and fraud risks of novel insect proteins within complex supply chains
Source: NPJ Sci Food. 2024 Jan 20;8:7. doi: 10.1038/s41538-023-00241-y (PMC10799884; doi:10.1038/s41538-023-00241-y)
Supplement: Supplementary file 1 — Supplementary Material [file 41538_2023_241_MOESM1_ESM.pdf]

Questionnaire for individuals along the insect supply chain:

Name of the company (if applicable):

Insect products relating to:

- ☐ Human food
- ☐ Animal feed
- ☐ Both

Country:

Name and position of the contact person:

Products and Supply Networks:

1. What is the function of you/your company within the insect protein industry (i.e. producer, trader, supplier)?

2. What does your general insect protein supply network look like i.e. your suppliers and who you are supplying to?

3. What insect species does your company handle? In what form are they received?

4. If the insect product(s) are processed, what kind of processing, where in the supply chain and/or by whom is the insect product(s) processed?

5. Does your supply network vary for different insect species such as crickets, mealworms? If so, can you please provide an example of how your supply network can vary for different species?

6. Does this supply network vary for different forms of the insects such as whole/raw vs. ground and dried? If so, can you please provide an example of how the supply network can vary for different forms of insects?

Food Safety and Allergenicity:

7. Are you aware of the rearing conditions or feed substrate of the insects used in your products? If so, what is the case?

8. When insect products arrive at your factories, are there any food safety or allergenicity assessments performed? What are the procedures?

9. If 'No' to previous question, does your supplier provide any information regarding this?

Potential avenues of food fraud:

11. Are you aware of food fraud within the insect protein industry?

12. What do you believe to be the impact (if any) of fraud on the edible insect supply network?

13. Are there any kinds of control measures in your company to guarantee the authentication of your products?

14. At what point in the insect protein supply chain do you believe to be most vulnerable to fraudulent activities?
